# How does genome instability affect lifespan?

**DOI:** 10.1111/j.1365-2443.2011.01519.x

**Published:** 2011-06

**Authors:** Takehiko Kobayashi

**Affiliations:** Division of Cytogenetics, National Institute of Genetics/The Graduate University for Advanced StudiesSOKENDAI, 1111 Yata, Mishima, Shizuoka 411-8540, Japan

## Abstract

The genome is composed not only of genes but also of several noncoding functional elements (NOCs/ncFE, here I use NOCs), such as transcriptional promoters, enhancers, replication origins, centromeres and telomeres. rDNA has both gene and NOC characteristics. Thus, the rDNA encodes ribosomal RNAs, components of the ribosomes, that account for approximately 80% of the total RNA in a cell. However, rDNA may also act as a NOC with respect to cellular senescence by limiting the number of times a cell can divide. Here, I discuss how rDNA might function as a NOC to influence life span in a manner analogous to telomeres.

## Introduction

The eukaryotic genome has many noncoding regions. In higher eukaryotic cells, most of these regions are occupied by retrotransposons, satellite DNA and other small repetitive elements. These regions used to be called ‘junk DNA’ and were presumed to have no meaningful role in the genome; however, recent studies have started, little by little, to show hidden functions (for review, see Alexander *et al.* 2010). In contrast to the presumptive junk DNA, there are several noncoding elements that are not associated with gene coding but are known to be functional; these are called NOCs (Ganley *et al.* 2005). Smaller NOCs, which range in size from tens to hundreds of DNA base pairs, can act as replication origins, replication fork barrier sites and recombination hotspots. Large NOCs function as centromeres or telomeres and can range in size from several hundred to thousands of kilobase pairs of DNA. Large NOCs sometimes include genes, e.g., tRNA genes in the centromere and some genes in the subtelomeric sequence, but it is not known whether the function of such genes is related to the functions of the NOCs.

The ribosomal RNA gene cluster (rDNA) occupies a large part of the genome and forms a unique repetitive region in a similar fashion to centromeres and telomeres. Of course, the ribosomal RNA gene itself is not a NOC, but it is known that the repeats have many untranscribed copies. In yeast and human cells, more than half of the repeats are silent (French *et al.* 2003). In this review, I introduce the rDNA as a kind of large NOCs and discuss its biological significance and implications.

## rDNA as the ‘king of the housekeeping genes’

**Figure 1:**
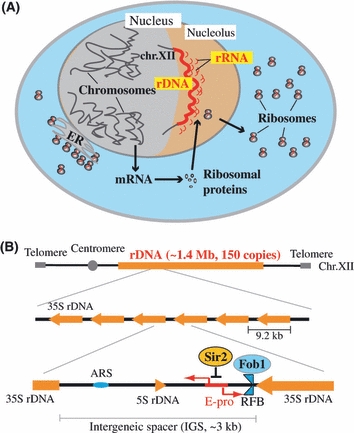
Structure of the budding yeast rDNA locus. (A) A cartoon of budding yeast. The rDNA is a tandem repeating array on chromosome XII in the nucleolus and producing ribosomal RNAs (rRNA) that are components of the ribosomes together with the ribosomal proteins. (B) Structure of yeast rDNA. A repeating unit (9.2 kb) has 5S and 35S rRNA genes and an intergenic spacer region. ARS and RFB are the replication origin and replication fork barrier site, respectively. E-pro is a bidirectional noncoding promoter that functions in the regulation of rDNA stability (copy number). The rDNA structure is broadly conserved from yeast to human; though, in the human genome, the 5S rDNA is found in independent arrays.

rDNA is the gene encoding ribosomal RNA (rRNA). In the budding yeast, *Saccharomyces cerevisiae*, rRNA is transcribed as 35S rRNA and then spliced into three mature rRNAs (18S, 5.8S and 26S). Together with a small, 5S, rRNA, which is independently transcribed from 35S rRNA, they form the skeletal framework of the ribosome. The ribosome, a complex of rRNAs and ribosomal proteins (rP), is a central player of gene expression, translating mRNA into protein (Fig. 1A). Ribosomes are present in very large numbers: rP accounts for approximately 50% of the total protein and rRNA represents approximately 80% of the total RNA in a cell (for review, see Warner 1999). To meet this huge demand, eukaryotic cells contain more than 100 copies of the gene for rRNA as a large cluster(s) on the genome (rDNA) and each cell produces approximately 2000 ribosomes per minute. rDNA is the most abundant gene in the cell. In *S. cerevisiae*, approximately 150 copies of rDNA are located on chromosome XII, occupying approximately 60% of the chromosome and approximately 10% of the whole genome (Fig. 1B). For these reasons, we can reasonably consider rDNA as the ‘king of the housekeeping genes’ in terms of function and quantity (for review, see Kobayashi 2011).

## Extracoding function of rDNA

**Figure 2:**
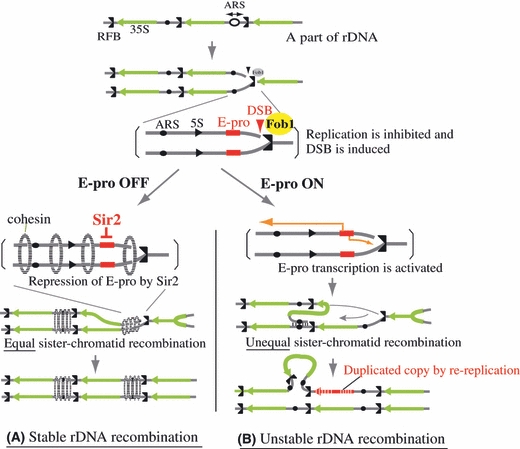
rDNA recombination for copy number change. In the S-phase of the cell cycle, by the function of Fob1, the DNA replication forks in the rDNA are inhibited at the RFB and DNA double-strand breaks (DSB) are induced. The broken ends are repaired by homologous recombination via sister chromatids. (A) In situations where copy number is normal, the silencing protein, Sir2, represses E-pro transcription, allowing the cohesin protein complex (dotted ellipse) to associate with the IGS. DSBs are repaired by equal sister-chromatid recombination, with no change in rDNA copy number. (B) In situations where copy number is reduced, Sir2 is not functioning and E-pro is activated. This E-pro transcription displaces cohesin from the IGS. The lack of cohesion means that unequal sister chromatids can be used as templates for the repair of DSBs, resulting in changes in rDNA copy number. The gray lines represent single chromatids (double-strand DNA).

rDNA is not only a crucial gene for basic cellular functions but also forms the largest repetitive domain in the genome. The repetitive nature of the rDNA region makes it highly recombinogenic and vulnerable to loss of copies after deleterious recombination events among the repeats. However, a gene amplification system ensures that the cell has a sufficient number of rDNA copies (Fig. 2B). Thus, the actual number of rDNA copies in a cell can vary both downward through deletion and upward by amplification (for review, see Kobayashi 2006). As a consequence, the rDNA region is the most unstable (fragile) part of the genome and this unusual characteristic affects cellular functions. Here, I term this aspect of rDNA as an extracoding function. As the instability of the rDNA region is independent of the 35S rDNA transcription (Kobayashi *et al.* 1998), the rDNA is essentially a large NOC similar to telomeres and centromeres. One of the prominent effects of genomic instability is on cellular senescence: instability has been shown to reduce life span in yeast and humans (Kobayashi 2008). Some human premature aging diseases, such as Werner, Bloom and Rothmund–Thomson syndromes, have mutations in DNA repair genes that maintain genomic stability (Ellis *et al.* 1995; Yu *et al.* 1996). In *S. cerevisiae*, defects in DNA repair dramatically reduce the replicative life span (Park *et al.* 1999). Interestingly, in this yeast, rDNA stability is known to determine the life span (Ganley *et al.* 2009).

## Telomeres as a lifespan determinant

Telomeres act as a lifespan determinant at the cellular and individual levels in some eukaryotes (Harley *et al.* 1990; Flores *et al.* 2008; Tomas-Loba *et al.* 2008). Telomeres are located at the ends of chromosomes; they protect the ends from degradation by nucleases and prevent connections with other chromosomal ends. The telomere is synthesized by a specific enzyme, telomerase, that contains template RNA to extend the end of the telomeric repeats (Greider & Blackburn 1985). As a replication fork cannot replicate the 3′-end of a telomere because the last primer that is required for lagging strand synthesis cannot be removed, this region is not synthesized. In humans, telomerase is expressed only in germ-line and stem cells. Therefore, in other cell types, such as differentiated cells, the chromosomal ends are shortened at successive cell divisions. In these cells, the length of the telomere is determined by the number of cell divisions since differentiation from the stem cells, which have telomerase activity. Telomere shortening can trigger the DNA-damage checkpoint response (DDR) and thereby cause arrest of the cell division cycle for repair (D'adda di Fagagna *et al.* 2003; Takai *et al.* 2003; Herbig *et al.* 2004). Thus, the shortening is believed to be a senescence signal to limit the number of cell divisions, i.e., ‘life span’, of each cell type (Bodnar *et al.* 1998).

## rDNA as a lifespan determinant

In the yeast *S. cerevisiae*, rDNA is known to affect life span, and two genes have been identified as being required for rDNA stability-dependent life span. One of these genes*, SIR2*, encoding the NAD^+^-dependent histone deacetylase, HDAC, is a repressor of the noncoding transcription that activates rDNA recombination as part of the amplification process (Figs 1B,2A). The defective *sir2Δ* gene causes an rDNA hyper-recombinogenic phenotype that has considerable copy number variation (Fig. 2B, Gottlieb & Esposito 1989; Kobayashi *et al.* 2004). The life span of the *sir2* mutant is approximately half that of the wild type (Kaeberlein *et al.* 1999). In contrast, the *FOB1* gene encodes a protein that inhibits the replication fork and causes double-strand breaks (DSBs) to trigger recombination in the rDNA (Kobayashi & Horiuchi 1996; Kobayashi *et al.* 2004). Fob1 is a key protein for the rDNA gene amplification system necessary to maintain the correct copy number (Fig. 2, Kobayashi *et al.* 1998). The *fob1* mutant, which represses rDNA recombination, has an approximately 60% longer life span compared with the wild type (Defossez *et al.* 1999; Takeuchi *et al.* 2003). In analogy to the behavior of telomere repeats, Fob1 could be considered to correspond to telomerase through its ability to extend the rDNA repeats, although its deletion phenotype is opposite that of telomerase in life span. The Fob1 protein localizes in the nucleolus and binds to a specific sequence at the 3′ end of the 35S rRNA gene in the rDNA (Kobayashi 2003). The double *SIR2*/*FOB1* mutant shows a similar phenotype to the *fob1* single mutant with respect to recombination and life span (Kobayashi *et al.* 2004), suggesting that recombination frequency (copy number instability) in the rDNA determines life span.

## Mechanism of rDNA-dependent lifespan determination

**Figure 3:**
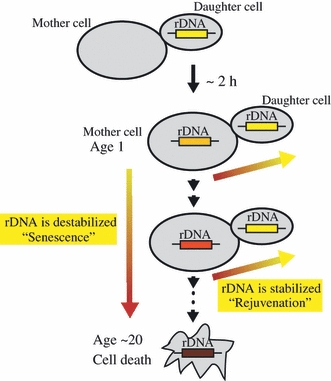
rDNA as an inducer of cellular senescence. The rDNA is one of the most unstable regions in the genome; therefore, it is most sensitive to exogeneous (e.g., UV) and endogeneous (e.g., NTP analogues) DNA-damaging factors. The rDNA senses such factors, becomes unstable, activates a DNA damage response and induces cellular senescence in the mother cell (down arrow). This rDNA instability is recovered in the daughter cell that can rejuvenate (horizontal arrows, Fig. 5).

The relationship between rDNA and life span was first shown by Guarente's group at MIT (Sinclair & Guarente 1997). They showed that ‘pop-out’ molecules (extrachromosomal rDNA circles, ERCs) derived from the rDNA by recombination accumulated in the mother cell and proposed that this accumulation induced senescence. Additionally, they found that other episomes (for example, plasmid vectors) that do not have a centromeric function also accumulated in the mother cell and might have similar effects on senescence. Many copies (>1000) of accumulated ERCs or episomes are thought to titrate factors that are required for the maintenance of ‘youthfulness’ in the mother cell; though, the identities of these factors are not yet known. Recently, we established a yeast strain in which DNA replication initiation activity in the rDNA was reduced (Ganley *et al.* 2009). In this strain, ERCs did not duplicate and, consequently, did not accumulate in the mother cell. However, in contrast to expectation, the life span of this strain was shortened. We analyzed the cause of this lifespan shortening and found that it depended on rDNA instability, which was only detectable in the mother cell (Fig. 3). As rDNA instability is known to increase the numbers of ERCs (Kaeberlein *et al.* 1999), we postulated that the instability was an upstream event of ERC accumulation and that it had a more direct impact on lifespan shortening. With regard to the relationships between non-rDNA episomes and life span, we found that episomes could also induce rDNA instability. They possibly titrate factors for chromosome maintenance (for example, topoisomerase and replication machinery), and rDNA is very sensitive to any shortage of these factors, which might result in rDNA instability and the shortening of the life span (Ide *et al.* 2007).

**Figure 4:**
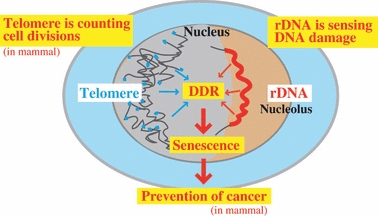
rDNA as a DNA damage sensor. Instability in the rDNA and telomeres activates DNA damage response and induces cellular senescence. The rDNA is sensing DNA damage and telomeres are counting cell divisions (in case of mammalian cells). The senescence is thought to be a system to kill cells before they become abnormal.

How does rDNA instability shorten life span? One possible explanation is that rDNA instability decreases the quality and quantity of ribosomes, thereby impairing cellular functions and, as a result, inducing cellular senescence (Kobayashi 2008). However, the Kennedy and Kaeberlein groups at Washington University found that deficiency in ribosomal proteins extends life span (Steffen *et al.* 2008). They speculated that the metabolic activity of mutants is reduced in a similar fashion as under calorie restriction, which is known to extent life span in many organisms including *S. cerevisiae* (Lin *et al.* 2000; Masoro 2005). Therefore, the ribosome deficiency model is unlikely to explain the effect of rDNA instability on life span. An alternative explanation is that the effect is related to the DDR. As mentioned in the telomere section previously, DDR is a system to repair DNA damage by inhibition of cell cycle progression and activation of the repair pathway. During cellular senescence, DDR is induced and inhibits cell division. DDR is also known to be induced by telomere shortening, and human cells with defective DDR show an extended life span (Shay *et al.* 1991; D'adda di Fagagna *et al.* 2003). As mentioned earlier, rDNA becomes increasingly unstable as the mother cell ages. If rDNA can act as an internal inducer of DDR, in an analogous fashion to telomeres, then it is possible that rDNA instability induces cellular senescence through DDR induction (Fig. 4). With regard to a mechanism for this postulated effect, I suggest that during the extended period of DDR-dependent cell cycle arrest, factors (currently unidentified) for the maintenance of youthfulness might show a reduction in their functions, resulting in the promotion of cellular senescence.

## Telomeres and rDNA have overlapping roles in cellular senescence

As described previously, there are reasons to suspect that rDNA is involved in cellular senescence and DDR in a similar manner to telomeres. Both of these regions are unstable parts of the genome: telomeres are shortened at successive cell divisions, whereas rDNAs undergo repeated recombination that escalates as the cell ages. However, the biological roles of these two regions are different. With regard to telomeres, their lengths are gradually reduced by cell division and, beyond a threshold limit value, cellular senescence is induced (D'adda di Fagagna *et al.* 2003; Takai *et al.* 2003; Herbig *et al.* 2004). As the rate of shortening is basically determined by the lengths of Okazaki fragments or RNA primers, the life span of the cell (i.e., the number of potential cell divisions) is effectively scheduled. Thus, the telomere ‘counts’ cell divisions to start senescence. In contrast, the stability of the rDNA is affected by internal and external DNA-damaging factors, e.g., UV, mutagens, NTP analogues and reactive oxygen species. DNA lesions produced by such factors inhibit replication forks and cause DSBs during the S-phase in the cell cycle. Of course, such factors induce damage not only in rDNA but also in other non-rDNA regions. In non-rDNA regions, DSBs are repaired by recombination with the sister chromatid (Kadyk & Hartwell 1992). However, in the case of rDNA, the broken end may recombine with the sister chromatid of neighboring homologous copies or its own strand because of the repetitive nature of the structure (Fig. 2B). Such unequal sister-chromatid recombination repair changes the copy number and entangles the repeats, resulting in a reduction in rDNA stability. Therefore, the rDNA region is more sensitive to DNA-damaging factors than non-rDNA regions. Because of this increased response to DNA-damaging factors, it is possible that the rDNA region can more rapidly induce a checkpoint control than any other genomic region. Thus, the rDNA might function as a ‘sensor’ for damage. In summary, both telomeres and rDNA induce cellular senescence, the former through a scheduled process and the latter by a sensory mechanism (Fig. 4).

## Biological meaning of senescence induced by telomeres or rDNA

To this point, I have discussed the mechanisms through which cellular senescence might be induced by telomeres or rDNA. Now, I wish to consider the biological implications of senescence, whether it results from metabolic pathways. We may obtain some insight into these implications by observation of long lifespan mutants. The *fob1* mutant, as mentioned earlier, has an approximately 60% longer life span than the wild type because of its stabilized rDNA. In wild-type yeast cells, mutations occur more frequently in old than in young cells (McMurray & Gottschling 2003). Analysis of the *fob1* mutant showed that the rate of accumulation of mutations was similar to that of the wild type. Therefore, as a result of longer life span, the frequency of mutated cells in the population increased. In human cells, mutations of DDR-related genes and/or forced expression of telomerase extend life span (Shay *et al.* 1991; Bodnar *et al.* 1998; Tomas-Loba *et al.* 2008). Both of these conditions result in an increase incidence of abnormal cells, such as cancer cells (González-Suárez *et al.* 2002; Canela *et al.* 2004). One possible role of cellular senescence might therefore be to suppress cellular abnormalities by limiting their life span. This function would be especially important in multicellular organisms to avoid cancer. For unicellular organisms, cellular senescence would help to maintain genetic stability in the population (Fig. 4).

## Reprogramming of aged cells

DNA damage–induced cellular senescence possibly occurs in most cells. However, there are some cell types that avoid senescence or that age very slowly, e.g., germ-line and stem cells. Neurons are long-lived cells that survive until the body dies, although they do not divide after the establishment of the neural network. In contrast, germ-line and stem cells duplicate their DNA and undergo cell division. Therefore, they have a need of mechanisms that prevent genomic instability as a result of repeated replication. Telomerase is expressed and telomere lengths are maintained in these cells (Flores *et al.* 2006). Moreover, stem cells have been suggested to contain a unique system to reduce the accumulation of mutation, the so-called immortal strand hypothesis (Cairns 1975; Rando 2007). When stem cells differentiate, the sister chromatid with the old DNA strand segregates to the stem cell and not to the differentiated cell. Theoretically, the old DNA strand will have fewer mutations because it is used as a template and has undergone fewer replication cycles. However, there is still considerable controversy regarding how such a nonrandom segregation mechanism might work with respect to maintaining genomic integrity in the stem cell (Lansdorp 2007).

**Figure 5:**
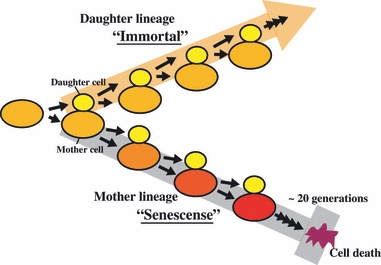
Asymmetrical cell division in budding yeast. Budding yeast divides asymmetrically. The mother (bigger) cell ages with each cell division, leading to senescence after approximately 20 cell cycles (lower ‘mother lineage’). However, the daughter cell (smaller) maintains the capability for division forever (upper ‘daughter lineage’). Yellow (small) and red (including orange and purple) cells indicate rejuvenated daughter and aging mother cells, respectively.

The budding yeast is a good model system for analyzing mechanisms that immortalize cells and counteract senescence. In mitosis, the yeast divides asymmetrically and produces two different cells, similar to stem cell differentiation (Fig. 5). One of these cells is the ‘mother cell’, which shows senescence and dies after approximately 20 cell divisions. The other cell is the ‘daughter cell’, which is budded from the mother cell. The genome of the daughter cell is reset and recovers the ability for cell division regardless of the senescence level of the mother cell. Daughter cells are so-called ‘immortal cells’. In other words, this asymmetric cell division is a rejuvenation process to maintain continuation of the species. Therefore, the daughter cell is the equivalent of progeny in higher organisms, although this generational change occurs after only one cell division. In terms of the mechanism of rejuvenation, telomere length does not seem to be involved. In budding yeast, telomerase is thought to be stably expressed in both mother and daughter cells and telomere shortening is not observed. In contrast, the stability of rDNA in the mother cell worsens with age. Interestingly, we recently found that daughter cells show the recovery of rDNA stability during cell division (Fig. 3, Ganley *et al.* 2009). This observation suggests a possible explanation for rejuvenation in the daughter cell: to reset senescence, the daughter cell has to reset rDNA instability. It will be interesting to determine how yeast restricts the repair of rDNA to the daughter cell.

## Conclusions

The telomere is a well-known lifespan determinant in eukaryotic cells. Here, I have discussed evidence that rDNA has overlapping roles with the telomere regarding cellular life spans. Both of these chromosomal regions issue an advance warning of genomic instability in the cell. They differ in that the telomere counts the number of cell divisions, whereas rDNA monitors how the genome is damaged (Fig. 4). Some organisms do not have typical telomeres, although all organisms have rDNA. This suggests that rDNA is the more common lifespan determinant. As rDNA is a vital gene encoding the ribosomal RNA, such an extracoding function (i.e., one not related to ribosomal RNA production) has not previously been given much consideration. It may be an inherent feature of this highly conserved housekeeping gene that it has an important role for cellular senescence in addition to RNA production. This role of rDNA ‘as a sensor’ is dependent on its instability, which is caused by the abundance of copies and its repetitive structure. Cells are known to possess many extra nontranscribed rDNA copies. Recently, we found that a yeast strain without theses extra copies showed genomic instability and became sensitive to DNA damage (Ide *et al.* 2010). We expect that further study of rDNA extracoding functions will show the relationship between rDNA and the control of damage sensitivity in cells and should aid in the development of new strategies for the prevention of cancers caused by genomic instability.
